# Odontological Society of Great Britain

**Published:** 1889-07

**Authors:** 


### SELECTIONS.

### Odontological Society of Great Britain.

MEETING OF MONDAY, APRIL 1, 1889. THE PRESIDENT, HENRY SEWILL, M.R.C.S., L.D. S., IN THE CHAIR.

Mr. Jonathan Hutchinson, F.R.S., read a paper upon

### SURGICAL DIAGNOSIS BY THE TEETH.

Referring to pyorrhœa alveolaris (Riggs' disease), a condition in which the gums recede and non-carious teeth are shed the author pointed out that an analogous disease—sycosis—affects the hair, in which a suppurative inflammation attacks the hair follicles and causes the hairs to drop out. Similarly the nails are in rare cases thrown off by reason of sycosis affecting their matrices. Sycosis of the beard, of the eyelashes (misnamed tinea tarsi, since no cryptogam exists), one and all have a similar pathogenesis, viz., a contagious inflammatory suppuration, the pus being carried from hair to hair and exciting fresh foci of suppuration. Although often occurring in weakly subjects, it was not necessarily confined to these; indeed, he regarded these forms of sycosis as local, and not constitutional conditions. The treatment seemed to lend color to this theory, for while tonics and general remedies did not ameliorate the disease, local epilation never failed. Comparing sycosis with pyorrhœa alveolaris, the lecturer insisted that the local inflammatory theory explained all the conditions. The purulent spots at the gum margins of the teeth infected one after the other, and so the disease gradually spread.

### MATERIALS USED FOR STOPPING TEETH.

Without for a moment disparaging the value of amalgams as a stopping material, Mr. Hutchinson believed that in some rare instances they were the cause of intractable and irritable sores upon the lips, gums, and cheeks. He spoke simply as an observer, not knowing the chemical composition of these stoppings, many of which he was told, were secret preparations; but he had certainly seen ulcers which refused to yield to treatment disappear after the removal of discolored amalgams. He had always been careful to eliminate the probability of roughness of the stopped teeth being the cause, and had never seen ulcers where gold fillings had been employed. He quoted a case of an American physician who presented a number of ulcers which several surgeons had pronounced syphilitic; the patient, however, stoutly maintained that he had never had syphilis or a symptom of it, and all his children were perfectly healthy. Mr. Hutchinson, finding that he had several amalgam fillings, which had been inserted some years before in America, ordered their removal, which being done, the ulcers rapidly disappeared.

SYPHILITIC TEETH.

Mr. Hutchinson said he had little new to say on this subject. The notched incisors were now generally admitted as evidence of hereditary syphilis. The upper centrals are the *test* teeth, and in no single instance had they misled the lecturer; but the markings on other teeth might be misleading, as they were often caused by the mercury given as a remedy for the syphilis, and were not due to the syphilis itself. In the majority of cases of hereditary syphilis the markings on the central incisors are present, but not always so. Of several members of the same family one or two might have the characteristic teeth, while the others escaped. He wished to draw attention to two points of clinical observation: (1) In all cases where interstitial keratitis occurs there are found malformed teeth, and those who have the *test* teeth characteristically marked have suffered from interstitial keratitis; (2) children who show signs of hereditary syphilis in the shape of phagedænic affections of the throat have no peculiarity of the teeth, and do not have interstitial keratitis.

The teeth affected by stomatitis, whether mercurial or other, were often mistaken for syphilitic teeth. Mercury given in infancy set up inflammation of the tooth sacs, and so led to deformity of the enamel. The test teeth in these cases are the first permanent molars, which might be compared with the premolars. Lamellar cataract, so commonly associated with stomatitic teeth, was due to convulsions. In adult life the presence of mercurial teeth was an assistance. Often the smallest dose of mercury would in these cases produce salivation, and the teeth revealing the idiosyncrasy would warn against its incautious use. The correlation of dental defects with errors in other structures had been brought before the society by Mr. Moon. Rat-like teeth were due to suppression of lateral denticles, and were often associated with micro-ophthalmos and deficiency in hair. Skin defects, however, are not always accompanied by tooth lesion. Referring to dentition in rickets, Mr. Hutchinson deprecated speaking of any definite rachitic teeth; the disease attacked the teeth as it did other structures.

The value of considering the teeth lay in answering the question, not, are the teeth present, but rather how and why did the teeth go? Some constitutional conditions would affect this, or in some cases local disease, as in pyorrhœa alveolaris. Again, tatar causing gum absorption often ran in families. In some cases it was due to excessive salivary secretion. The lecturer did not consider gout, as such, produced any typical teeth. As long ago as Graves, tooth grinding had been shown to be common among the gouty, and this practice lead to wearing down the teeth.

DISCUSSION.

The president pointed out that, although Mr. Hutchinson saw a few cases in which amalgam fillings had presumably produced sore tongue, yet the profession saw thousands of cases where amalgams properly inserted produced no untoward results. He thought the paper would lead to important results as regards the treatment of pyorrhœa alveolaris.

Several other speakers also insisted upon the harmlessness of good amalgams if properly finished off in the mouth so as to leave no rough edges.

Mr. C. S. Tomes remarked that, whereas the unsymmetrical manner in which pyorrhœa alveolaris attacked the teeth was contrary to the theory of local infection, yet the inability to cure the disease until the teeth were removed supported it.

Mr. Charters White felt that as the roots were dead and necrosed, and nature was evidently intending to throw off the teeth, it would be unscientific to apply treatment except in so far as would assist the natural process.

Mr. F. Newland Pedley, while deprecating the attack upon amalgams, pointed out that in unskilled hands large amalgam fillings chipped, and so might leave ragged edges, and this was the fault of the filler rather than of the filling.

Mr. Storer Bennett thought pyorrhœa alveolaris was frequently associated with depressed conditions of vitality. Animals in captivity were unfavorably placed as regard bodily vigor, and they suffered from this complaint.—*Medical Press and Circular.*

